# 2-(4-Meth­oxy­benz­yl)-4,6-diphenyl-2,5-diaza­bicyclo­[2.2.2]oct-5-en-3-one

**DOI:** 10.1107/S1600536811012165

**Published:** 2011-04-07

**Authors:** Jo Alen, Liliana Dobrzańska, Luc Van Meervelt, Wim M. De Borggraeve

**Affiliations:** a Molecular Design & Synthesis, Department of Chemistry, Katholieke Universiteit Leuven, Celestijnenlaan 200F – box 2404, B-3001 Leuven, Belgium; b Biomolecular Architecture, Department of Chemistry, Katholieke Universiteit Leuven, Celestijnenlaan 200F – box 2404, B-3001 Leuven, Belgium

## Abstract

In the crystal structure of the title compound, C_26_H_24_N_2_O_2_, weak inter­molecular C—H⋯π inter­actions involving the benzene of the *p*-methoxy benzyl group and one of the phenyl rings result in the formation of chains consisting of alternating enanti­omers. Weak C—H ⋯O inter­actions with the methoxy O atom lead to the formation of layers, which are inter­linked by further C—H⋯O inter­actions into a three-dimensional assembly.

## Related literature

For our studies on pyrazinone chemistry, see: De Borggraeve *et al.* (2004 ▶); Azzam *et al.* (2004 ▶); Alen *et al.* (2007*a* ▶); Rombouts *et al.* (2003 ▶). For a crystal structure with a 2,5-diaza­bicyclo­[2.2.2]oct-5-en-3-one core, see: Rusinov *et al.* (2009 ▶). For crystal structures with a similar 2,5-diaza­bicyclo­[2.2.2]octane-3,6-dione core, see: Alen *et al.* (2007*b* ▶); Holl *et al.* (2008 ▶).
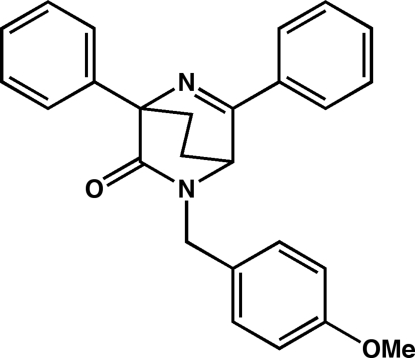


## Experimental

### 

#### Crystal data

C_26_H_24_N_2_O_2_
*M* *_r_* = 396.47
Triclinic, 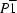

*a* = 6.2770 (1) Å
*b* = 11.5684 (2) Å
*c* = 14.1443 (2) Å
α = 85.497 (1)°
β = 89.900 (1)°
γ = 76.144 (1)°
*V* = 993.97 (3) Å^3^
*Z* = 2
Cu *K*α radiation
μ = 0.67 mm^−1^
*T* = 100 K
0.34 × 0.18 × 0.15 mm

#### Data collection

Bruker SMART 6000 diffractometer
Absorption correction: multi-scan (*SADABS*; Sheldrick, 1997 ▶) *T*_min_ = 0.805, *T*_max_ = 0.907
10093 measured reflections
3473 independent reflections
2894 reflections with *I* > 2σ(*I*)
*R*_int_ = 0.028

#### Refinement

*R*[*F* ^2^ > 2σ(*F* ^2^)] = 0.039
*wR*(*F* ^2^) = 0.105
*S* = 1.05
3473 reflections
272 parameters
H-atom parameters constrained
Δρ_max_ = 0.25 e Å^−3^
Δρ_min_ = −0.27 e Å^−3^

### 

Data collection: *SMART* (Bruker, 2001 ▶); cell refinement: *SAINT* (Bruker, 2002 ▶); data reduction: *SAINT*; program(s) used to solve structure: *SHELXS97* (Sheldrick, 2008 ▶); program(s) used to refine structure: *SHELXL97* (Sheldrick, 2008 ▶); molecular graphics: *Mercury* (Macrae *et al.*, 2008 ▶); software used to prepare material for publication: *SHELXL97*.

## Supplementary Material

Crystal structure: contains datablocks I, global. DOI: 10.1107/S1600536811012165/hg5019sup1.cif

Structure factors: contains datablocks I. DOI: 10.1107/S1600536811012165/hg5019Isup2.hkl

Additional supplementary materials:  crystallographic information; 3D view; checkCIF report

## Figures and Tables

**Table 1:** Hydrogen-bond geometry (Å, °) *Cg*1 and *Cg*2 are the centroids of the C22–C27 and C9–C14 rings, respectively.

*D*—H⋯*A*	*D*—H	H⋯*A*	*D*⋯*A*	*D*—H⋯*A*
C8—H8*A*⋯*Cg*2^i^	0.99	2.96	3.906 (2)	159
C21—H21*A*⋯*Cg*1^ii^	0.99	2.56	3.411 (2)	144
C19—H19⋯O28^iii^	0.95	2.51	3.457 (2)	172
C13—H13⋯O30^iv^	0.95	2.56	3.368 (2)	143
C29—H29*A*⋯O30^v^	0.98	2.50	3.383 (2)	150

Symmetry codes: (i) 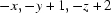; (ii) 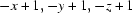; (iii) 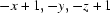; (iv) 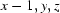; (v) 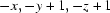.

**Fig. 1.**
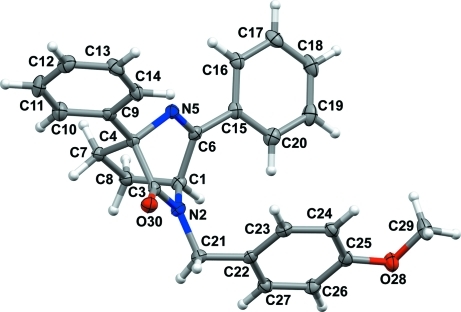
The molecular structure of the title molecule; displacement ellipsoids are drawn at the 50% probability level.

**Fig. 2.**
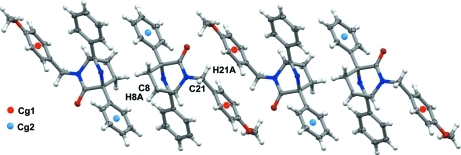
Fragment of the chain formed by C—H···π interactions; red centroids derive from C22–C27 ring (Cg1), blue from C9–C14 (Cg2).

**Fig. 3.**
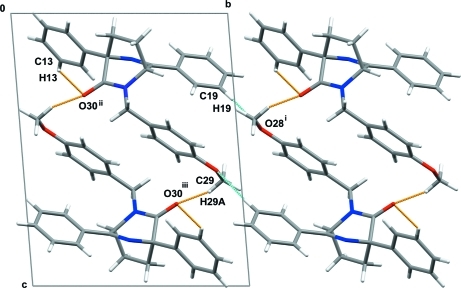
Representation of the packing viewed down the a axis; weak C19—H19···O28 interactions facilitating the formation of layers are indicated by blue dashed lines; C13—H13···O30 and C29—H29A···O30 interactions stabilizing the three-dimensional assembly are presented in orange. Symmetry codes are listed in Table 1.

## supplementary crystallographic information

### Comment

During the course of our studies on 3,5-dichloropyrazinones (Azzam *et al.*, 2004; Alen *et al.*, 2007*a*) and their conversion to aminopiperidinone carboxylate systems (Rombouts *et al.*, 2003; De Borggraeve *et al.*, 2004), we have isolated the title compound. Although quite a few studies deal with bicyclo[2.2.2]octane systems, only one structure with the 2,5-diazabicyclo[2.2.2]oct-5-en-3-one core (Rusinov *et al.*, 2009) has been reported till now. Quite a few structures contain the similar 2,5-diazabicyclo[2.2.2]octane-3,6-dione core. Of those, two have very close resemblance to the title molecule due to a benzyl substituent on N4. One of those was obtained by us (Alen *et al.*, 2007*b*) and the other one was published by Wünsch and co-workers (Holl *et al.*, 2008).

The presented structure crystallizes in the triclinic space group *P*1 with one molecule in the asymmetric unit (Fig. 1). All three aromatic rings participate in weak C—H···π interactions, acting as a donor (C15–C20) or acceptors (C9–C14 and C22–C27). Intramolecular interactions C20—H20···*Cg*1, (where *Cg*1 is the centroid of the C22–C27 ring; the C20···*Cg*1 distance is 3.790 (2) Å, and the C20—H20···*Cg*1 angle is 149°) influence the orientation of these two rings towards each other. The dihedral angle between their planes is 52.75 (4)°. Rings C9–C14 and C22–C27, with dihedral angles 12.77 (9)° between their corresponding planes and -117.75 (1)° between C9–C4–C21–C22, are involved in weak intermolecular C—H···π interactions. These interactions, namely C8—H8A···*Cg*2^i^ (where *Cg*2 is the centroid of C9–C14, the C8···*Cg*2 distance is 3.906 (2) Å; symmetry operation (i): -*x*,1 - *y*,2 - *z*) and C21—H21A···*Cg*1^ii^ (the C21···*Cg*1 distance is 3.411 (2) Å; symmetry code (ii): 1 - *x*,1 - *y*,1 - *z*), lead to the formation of chains of alternating enantiomers along [-1 0 1] (Fig. 2).These chains are interlinked by C19—H19···O28 interactions to form layers, which are expanded in the third dimension through a number of C—H···O interactions involving O30 (Fig. 3, Table 1).

### Experimental

1-(4-methoxybenzyl)-3,5-diphenylpyrazin-2(1*H*)-one (5 mmol) was dissolved in toluene and heated at 145 °C in a stainless steel bomb under ethene pressure (35 atm) for 4 h. The progress of the Diels-Alder cycloaddition was monitored on TLC, by the disappearance of the starting pyrazinone. After evaporation of the solvent, the crude residue was purified by column chromatography to yield the title compound. Single crystals suitable for X-ray diffraction were obtained by slow evaporation from a chloroform solution.

### Refinement

All H atoms were positioned geometrically (C—H = 0.95, 0.98, 0.99 and 1 Å) and constrained to ride on their parent atoms with *U*_iso_(H) values set at 1.2 *x U*_eq_(C) and 1.5 *x U*_eq_(methyl-C).

### Crystal data

**Table:**

C_26_H_24_N_2_O_2_	*Z* = 2
*M**_r_* = 396.47	*F*(000) = 420
Triclinic, *P*1	*D*_x_ = 1.325 Mg m^−^^3^
Hall symbol: -P 1	Cu *K*α radiation, λ = 1.54178 Å
*a* = 6.2770 (1) Å	Cell parameters from 3657 reflections
*b* = 11.5684 (2) Å	θ = 4.0–68.4°
*c* = 14.1443 (2) Å	µ = 0.67 mm^−^^1^
α = 85.497 (1)°	*T* = 100 K
β = 89.900 (1)°	Block, colorless
γ = 76.144 (1)°	0.34 × 0.18 × 0.15 mm
*V* = 993.97 (3) Å^3^	

### Data collection

**Table:**

Bruker SMART 6000 diffractometer	3473 independent reflections
Radiation source: fine-focus sealed tube	2894 reflections with *I* > 2σ(*I*)
crossed Göbel mirrors	*R*_int_ = 0.028
ω and φ scans	θ_max_ = 68.5°, θ_min_ = 4.0°
Absorption correction: multi-scan (*SADABS*; Sheldrick, 1997)	*h* = −7→7
*T*_min_ = 0.805, *T*_max_ = 0.907	*k* = −13→13
10093 measured reflections	*l* = −16→16

### Refinement

**Table:**

Refinement on *F*^2^	Primary atom site location: structure-invariant direct methods
Least-squares matrix: full	Secondary atom site location: difference Fourier map
*R*[*F*^2^ > 2σ(*F*^2^)] = 0.039	Hydrogen site location: inferred from neighbouring sites
*wR*(*F*^2^) = 0.105	H-atom parameters constrained
*S* = 1.05	*w* = 1/[σ^2^(*F*_o_^2^) + (0.0591*P*)^2^ + 0.2042*P*] where *P* = (*F*_o_^2^ + 2*F*_c_^2^)/3
3473 reflections	(Δ/σ)_max_ < 0.001
272 parameters	Δρ_max_ = 0.25 e Å^−^^3^
0 restraints	Δρ_min_ = −0.27 e Å^−^^3^

### Special details

**Table:**

Geometry. All e.s.d.'s (except the e.s.d. in the dihedral angle between two l.s. planes) are estimated using the full covariance matrix. The cell e.s.d.'s are taken into account individually in the estimation of e.s.d.'s in distances, angles and torsion angles; correlations between e.s.d.'s in cell parameters are only used when they are defined by crystal symmetry. An approximate (isotropic) treatment of cell e.s.d.'s is used for estimating e.s.d.'s involving l.s. planes.
Refinement. Refinement of *F*^2^ against ALL reflections. The weighted *R*-factor *wR* and goodness of fit *S* are based on *F*^2^, conventional *R*-factors *R* are based on *F*, with *F* set to zero for negative *F*^2^. The threshold expression of *F*^2^ > σ(*F*^2^) is used only for calculating *R*-factors(gt) *etc*. and is not relevant to the choice of reflections for refinement. *R*-factors based on *F*^2^ are statistically about twice as large as those based on *F*, and *R*- factors based on ALL data will be even larger.

### Fractional atomic coordinates and isotropic or equivalent isotropic displacement parameters (Å^2^)

**Table:**

	*x*	*y*	*z*	*U*_iso_*/*U*_eq_	
C1	0.3365 (2)	0.39618 (12)	0.79614 (10)	0.0184 (3)	
H1	0.4452	0.3253	0.7747	0.022*	
N2	0.30988 (19)	0.50050 (10)	0.72762 (8)	0.0182 (3)	
C3	0.1528 (2)	0.59721 (12)	0.74710 (9)	0.0169 (3)	
C4	0.0393 (2)	0.57044 (12)	0.84081 (9)	0.0162 (3)	
N5	−0.04137 (19)	0.46034 (10)	0.83215 (8)	0.0167 (3)	
C6	0.1113 (2)	0.37223 (12)	0.80940 (9)	0.0167 (3)	
C7	0.2259 (2)	0.54041 (12)	0.91838 (9)	0.0181 (3)	
H7B	0.2907	0.6099	0.9226	0.022*	
H7A	0.1645	0.5227	0.9809	0.022*	
C8	0.4036 (2)	0.43205 (13)	0.89272 (10)	0.0195 (3)	
H8A	0.4150	0.3651	0.9420	0.023*	
H8B	0.5477	0.4527	0.8881	0.023*	
C9	−0.1499 (2)	0.67346 (12)	0.86188 (10)	0.0170 (3)	
C10	−0.1369 (3)	0.74953 (13)	0.93157 (10)	0.0242 (3)	
H10	−0.0042	0.7384	0.9670	0.029*	
C11	−0.3152 (3)	0.84182 (13)	0.95039 (11)	0.0278 (4)	
H11	−0.3041	0.8920	0.9992	0.033*	
C12	−0.5084 (3)	0.86085 (13)	0.89838 (11)	0.0252 (3)	
H12	−0.6301	0.9239	0.9113	0.030*	
C13	−0.5227 (2)	0.78717 (13)	0.82731 (11)	0.0252 (3)	
H13	−0.6542	0.8000	0.7907	0.030*	
C14	−0.3449 (2)	0.69462 (12)	0.80964 (11)	0.0219 (3)	
H14	−0.3566	0.6445	0.7608	0.026*	
C15	0.0681 (2)	0.25323 (12)	0.79874 (10)	0.0184 (3)	
C16	−0.1056 (2)	0.22043 (12)	0.84705 (10)	0.0200 (3)	
H16	−0.1934	0.2741	0.8872	0.024*	
C17	−0.1512 (2)	0.11049 (13)	0.83698 (11)	0.0256 (3)	
H17	−0.2689	0.0889	0.8705	0.031*	
C18	−0.0242 (3)	0.03196 (13)	0.77777 (12)	0.0282 (4)	
H18	−0.0539	−0.0439	0.7714	0.034*	
C19	0.1452 (3)	0.06430 (13)	0.72808 (11)	0.0273 (4)	
H19	0.2291	0.0116	0.6862	0.033*	
C20	0.1933 (2)	0.17386 (13)	0.73923 (10)	0.0230 (3)	
H20	0.3122	0.1947	0.7061	0.028*	
C21	0.4620 (2)	0.49909 (13)	0.64896 (10)	0.0200 (3)	
H21B	0.6110	0.4935	0.6746	0.024*	
H21A	0.4168	0.5750	0.6086	0.024*	
C22	0.4685 (2)	0.39600 (12)	0.58928 (9)	0.0185 (3)	
C23	0.2800 (2)	0.38246 (12)	0.54412 (10)	0.0200 (3)	
H23	0.1450	0.4391	0.5516	0.024*	
C24	0.2841 (2)	0.28809 (12)	0.48822 (10)	0.0206 (3)	
H24	0.1534	0.2800	0.4584	0.025*	
C25	0.4826 (2)	0.20556 (12)	0.47653 (10)	0.0204 (3)	
C26	0.6727 (2)	0.21824 (13)	0.52098 (10)	0.0215 (3)	
H26	0.8082	0.1624	0.5128	0.026*	
C27	0.6647 (2)	0.31205 (13)	0.57704 (10)	0.0204 (3)	
H27	0.7950	0.3193	0.6076	0.024*	
O28	0.50771 (17)	0.10994 (9)	0.42322 (7)	0.0259 (3)	
C29	0.3127 (3)	0.08362 (14)	0.38884 (12)	0.0289 (4)	
H29B	0.2193	0.0710	0.4424	0.043*	
H29C	0.3514	0.0112	0.3547	0.043*	
H29A	0.2334	0.1506	0.3458	0.043*	
O30	0.10832 (16)	0.69324 (8)	0.69944 (7)	0.0208 (2)	

### Atomic displacement parameters (Å^2^)

**Table:**

	*U*^11^	*U*^22^	*U*^33^	*U*^12^	*U*^13^	*U*^23^
C1	0.0169 (7)	0.0183 (7)	0.0186 (7)	−0.0017 (5)	0.0005 (5)	−0.0013 (5)
N2	0.0193 (6)	0.0193 (6)	0.0159 (6)	−0.0043 (5)	0.0026 (5)	−0.0015 (5)
C3	0.0166 (7)	0.0202 (7)	0.0155 (7)	−0.0064 (5)	−0.0013 (5)	−0.0041 (5)
C4	0.0177 (7)	0.0169 (7)	0.0151 (7)	−0.0060 (5)	−0.0002 (5)	−0.0025 (5)
N5	0.0178 (6)	0.0171 (6)	0.0156 (6)	−0.0047 (5)	−0.0003 (4)	−0.0018 (4)
C6	0.0178 (7)	0.0186 (7)	0.0126 (6)	−0.0026 (5)	−0.0012 (5)	0.0002 (5)
C7	0.0174 (7)	0.0235 (7)	0.0142 (7)	−0.0069 (6)	−0.0005 (5)	−0.0010 (5)
C8	0.0170 (8)	0.0245 (7)	0.0169 (7)	−0.0054 (6)	−0.0006 (5)	0.0002 (6)
C9	0.0189 (8)	0.0159 (7)	0.0171 (7)	−0.0065 (5)	0.0026 (5)	0.0001 (5)
C10	0.0262 (8)	0.0232 (8)	0.0229 (8)	−0.0042 (6)	−0.0026 (6)	−0.0045 (6)
C11	0.0383 (10)	0.0211 (8)	0.0228 (8)	−0.0030 (6)	−0.0013 (7)	−0.0077 (6)
C12	0.0263 (8)	0.0164 (7)	0.0304 (8)	−0.0001 (6)	0.0059 (6)	−0.0030 (6)
C13	0.0192 (8)	0.0201 (7)	0.0363 (9)	−0.0044 (6)	−0.0024 (6)	−0.0030 (6)
C14	0.0225 (8)	0.0173 (7)	0.0268 (8)	−0.0049 (6)	−0.0013 (6)	−0.0070 (6)
C15	0.0187 (8)	0.0172 (7)	0.0177 (7)	−0.0015 (5)	−0.0055 (5)	−0.0004 (5)
C16	0.0176 (8)	0.0190 (7)	0.0225 (7)	−0.0023 (5)	−0.0050 (5)	−0.0021 (6)
C17	0.0198 (8)	0.0238 (8)	0.0333 (8)	−0.0064 (6)	−0.0054 (6)	0.0003 (6)
C18	0.0304 (9)	0.0172 (7)	0.0365 (9)	−0.0043 (6)	−0.0121 (7)	−0.0033 (6)
C19	0.0312 (9)	0.0199 (7)	0.0274 (8)	0.0024 (6)	−0.0051 (6)	−0.0074 (6)
C20	0.0232 (8)	0.0208 (7)	0.0225 (7)	−0.0004 (6)	−0.0017 (6)	−0.0013 (6)
C21	0.0198 (8)	0.0227 (7)	0.0189 (7)	−0.0074 (6)	0.0040 (5)	−0.0034 (6)
C22	0.0214 (8)	0.0212 (7)	0.0140 (7)	−0.0074 (6)	0.0023 (5)	0.0002 (5)
C23	0.0190 (8)	0.0207 (7)	0.0196 (7)	−0.0034 (6)	0.0018 (5)	−0.0006 (6)
C24	0.0209 (8)	0.0230 (7)	0.0193 (7)	−0.0082 (6)	−0.0012 (6)	−0.0005 (6)
C25	0.0268 (8)	0.0184 (7)	0.0171 (7)	−0.0075 (6)	0.0019 (6)	−0.0020 (5)
C26	0.0192 (8)	0.0205 (7)	0.0235 (7)	−0.0021 (6)	0.0019 (6)	−0.0026 (6)
C27	0.0187 (8)	0.0239 (7)	0.0192 (7)	−0.0069 (6)	−0.0007 (5)	−0.0008 (6)
O28	0.0269 (6)	0.0229 (5)	0.0293 (6)	−0.0059 (4)	−0.0015 (4)	−0.0096 (4)
C29	0.0319 (9)	0.0225 (8)	0.0340 (9)	−0.0083 (7)	−0.0084 (7)	−0.0067 (7)
O30	0.0241 (6)	0.0189 (5)	0.0191 (5)	−0.0053 (4)	0.0002 (4)	0.0013 (4)

### Geometric parameters (Å, °)

**Table:**

C1—N2	1.4641 (18)	C15—C20	1.394 (2)
C1—C6	1.5129 (19)	C15—C16	1.398 (2)
C1—C8	1.5460 (18)	C16—C17	1.386 (2)
C1—H1	1.0000	C16—H16	0.9500
N2—C3	1.3485 (18)	C17—C18	1.390 (2)
N2—C21	1.4633 (17)	C17—H17	0.9500
C3—O30	1.2252 (17)	C18—C19	1.383 (2)
C3—C4	1.5489 (19)	C18—H18	0.9500
C4—N5	1.4922 (17)	C19—C20	1.392 (2)
C4—C9	1.5149 (19)	C19—H19	0.9500
C4—C7	1.5641 (18)	C20—H20	0.9500
N5—C6	1.2820 (18)	C21—C22	1.5070 (19)
C6—C15	1.4843 (19)	C21—H21B	0.9900
C7—C8	1.5322 (19)	C21—H21A	0.9900
C7—H7B	0.9900	C22—C23	1.393 (2)
C7—H7A	0.9900	C22—C27	1.394 (2)
C8—H8A	0.9900	C23—C24	1.393 (2)
C8—H8B	0.9900	C23—H23	0.9500
C9—C10	1.387 (2)	C24—C25	1.395 (2)
C9—C14	1.391 (2)	C24—H24	0.9500
C10—C11	1.391 (2)	C25—O28	1.3653 (17)
C10—H10	0.9500	C25—C26	1.393 (2)
C11—C12	1.382 (2)	C26—C27	1.385 (2)
C11—H11	0.9500	C26—H26	0.9500
C12—C13	1.384 (2)	C27—H27	0.9500
C12—H12	0.9500	O28—C29	1.4250 (18)
C13—C14	1.387 (2)	C29—H29B	0.9800
C13—H13	0.9500	C29—H29C	0.9800
C14—H14	0.9500	C29—H29A	0.9800
			
N2—C1—C6	106.68 (11)	C9—C14—H14	119.3
N2—C1—C8	107.79 (11)	C20—C15—C16	118.74 (13)
C6—C1—C8	106.12 (11)	C20—C15—C6	121.48 (13)
N2—C1—H1	112.0	C16—C15—C6	119.75 (12)
C6—C1—H1	112.0	C17—C16—C15	120.75 (14)
C8—C1—H1	112.0	C17—C16—H16	119.6
C3—N2—C21	123.95 (12)	C15—C16—H16	119.6
C3—N2—C1	115.85 (11)	C16—C17—C18	119.89 (15)
C21—N2—C1	119.96 (11)	C16—C17—H17	120.1
O30—C3—N2	125.58 (12)	C18—C17—H17	120.1
O30—C3—C4	124.51 (12)	C19—C18—C17	119.93 (14)
N2—C3—C4	109.89 (11)	C19—C18—H18	120.0
N5—C4—C9	110.11 (11)	C17—C18—H18	120.0
N5—C4—C3	108.07 (10)	C18—C19—C20	120.23 (14)
C9—C4—C3	111.77 (11)	C18—C19—H19	119.9
N5—C4—C7	107.65 (10)	C20—C19—H19	119.9
C9—C4—C7	113.68 (11)	C19—C20—C15	120.42 (15)
C3—C4—C7	105.25 (11)	C19—C20—H20	119.8
C6—N5—C4	112.30 (11)	C15—C20—H20	119.8
N5—C6—C15	121.31 (12)	N2—C21—C22	112.07 (11)
N5—C6—C1	116.26 (12)	N2—C21—H21B	109.2
C15—C6—C1	122.43 (12)	C22—C21—H21B	109.2
C8—C7—C4	109.57 (11)	N2—C21—H21A	109.2
C8—C7—H7B	109.8	C22—C21—H21A	109.2
C4—C7—H7B	109.8	H21B—C21—H21A	107.9
C8—C7—H7A	109.8	C23—C22—C27	118.15 (13)
C4—C7—H7A	109.8	C23—C22—C21	121.17 (12)
H7B—C7—H7A	108.2	C27—C22—C21	120.68 (12)
C7—C8—C1	107.33 (11)	C24—C23—C22	121.73 (13)
C7—C8—H8A	110.2	C24—C23—H23	119.1
C1—C8—H8A	110.2	C22—C23—H23	119.1
C7—C8—H8B	110.2	C23—C24—C25	119.11 (13)
C1—C8—H8B	110.2	C23—C24—H24	120.4
H8A—C8—H8B	108.5	C25—C24—H24	120.4
C10—C9—C14	117.80 (13)	O28—C25—C26	115.66 (13)
C10—C9—C4	122.53 (13)	O28—C25—C24	124.54 (13)
C14—C9—C4	119.66 (12)	C26—C25—C24	119.80 (13)
C9—C10—C11	121.05 (14)	C27—C26—C25	120.19 (13)
C9—C10—H10	119.5	C27—C26—H26	119.9
C11—C10—H10	119.5	C25—C26—H26	119.9
C12—C11—C10	120.35 (14)	C26—C27—C22	121.01 (13)
C12—C11—H11	119.8	C26—C27—H27	119.5
C10—C11—H11	119.8	C22—C27—H27	119.5
C11—C12—C13	119.34 (14)	C25—O28—C29	117.06 (11)
C11—C12—H12	120.3	O28—C29—H29B	109.5
C13—C12—H12	120.3	O28—C29—H29C	109.5
C12—C13—C14	119.95 (14)	H29B—C29—H29C	109.5
C12—C13—H13	120.0	O28—C29—H29A	109.5
C14—C13—H13	120.0	H29B—C29—H29A	109.5
C13—C14—C9	121.49 (13)	H29C—C29—H29A	109.5
C13—C14—H14	119.3		
			
C6—C1—N2—C3	51.84 (15)	C4—C9—C10—C11	−178.69 (13)
C8—C1—N2—C3	−61.78 (15)	C9—C10—C11—C12	−1.2 (2)
C6—C1—N2—C21	−133.60 (12)	C10—C11—C12—C13	0.0 (2)
C8—C1—N2—C21	112.78 (13)	C11—C12—C13—C14	0.7 (2)
C21—N2—C3—O30	4.4 (2)	C12—C13—C14—C9	−0.1 (2)
C1—N2—C3—O30	178.72 (12)	C10—C9—C14—C13	−1.1 (2)
C21—N2—C3—C4	−174.13 (11)	C4—C9—C14—C13	179.33 (13)
C1—N2—C3—C4	0.19 (16)	N5—C6—C15—C20	153.12 (13)
O30—C3—C4—N5	126.98 (13)	C1—C6—C15—C20	−28.10 (19)
N2—C3—C4—N5	−54.46 (14)	N5—C6—C15—C16	−25.04 (19)
O30—C3—C4—C9	5.66 (18)	C1—C6—C15—C16	153.74 (13)
N2—C3—C4—C9	−175.79 (11)	C20—C15—C16—C17	0.8 (2)
O30—C3—C4—C7	−118.21 (14)	C6—C15—C16—C17	178.97 (13)
N2—C3—C4—C7	60.35 (13)	C15—C16—C17—C18	−0.4 (2)
C9—C4—N5—C6	176.59 (11)	C16—C17—C18—C19	−0.9 (2)
C3—C4—N5—C6	54.24 (14)	C17—C18—C19—C20	1.9 (2)
C7—C4—N5—C6	−58.98 (14)	C18—C19—C20—C15	−1.5 (2)
C4—N5—C6—C15	178.84 (11)	C16—C15—C20—C19	0.2 (2)
C4—N5—C6—C1	−0.01 (16)	C6—C15—C20—C19	−177.95 (13)
N2—C1—C6—N5	−53.75 (15)	C3—N2—C21—C22	−129.16 (13)
C8—C1—C6—N5	61.00 (15)	C1—N2—C21—C22	56.75 (16)
N2—C1—C6—C15	127.41 (13)	N2—C21—C22—C23	59.17 (17)
C8—C1—C6—C15	−117.83 (13)	N2—C21—C22—C27	−121.48 (14)
N5—C4—C7—C8	55.29 (14)	C27—C22—C23—C24	0.2 (2)
C9—C4—C7—C8	177.54 (11)	C21—C22—C23—C24	179.53 (12)
C3—C4—C7—C8	−59.81 (13)	C22—C23—C24—C25	−0.5 (2)
C4—C7—C8—C1	2.67 (14)	C23—C24—C25—O28	−179.64 (13)
N2—C1—C8—C7	56.69 (14)	C23—C24—C25—C26	0.3 (2)
C6—C1—C8—C7	−57.31 (13)	O28—C25—C26—C27	−179.72 (12)
N5—C4—C9—C10	134.06 (13)	C24—C25—C26—C27	0.3 (2)
C3—C4—C9—C10	−105.81 (15)	C25—C26—C27—C22	−0.7 (2)
C7—C4—C9—C10	13.18 (18)	C23—C22—C27—C26	0.5 (2)
N5—C4—C9—C14	−46.41 (16)	C21—C22—C27—C26	−178.88 (13)
C3—C4—C9—C14	73.72 (15)	C26—C25—O28—C29	170.61 (13)
C7—C4—C9—C14	−167.29 (12)	C24—C25—O28—C29	−9.5 (2)
C14—C9—C10—C11	1.8 (2)		

### Hydrogen-bond geometry (Å, °)

**Table:**

Cg1 and Cg2 are the centroids of the C22–C27 and C9–C14 rings, respectively.

**Table:**

*D*—H···*A*	*D*—H	H···*A*	*D*···*A*	*D*—H···*A*
C19—H19···O28^i^	0.95	2.51	3.457 (2)	172
C13—H13···O30^ii^	0.95	2.56	3.368 (2)	143
C29—H29A···O30^iii^	0.98	2.50	3.383 (2)	150
C8—H8A···Cg2^iv^	0.99	2.96	3.906 (2)	159
C21—H21A···Cg1^v^	0.99	2.56	3.411 (2)	144

Symmetry codes: (i) −*x*+1, −*y*, −*z*+1; (ii) *x*−1, *y*, *z*; (iii) −*x*, −*y*+1, −*z*+1; (iv) −*x*, −*y*+1, −*z*+2; (v) −*x*+1, −*y*+1, −*z*+1.
